# Prosocial behavior in students with intellectual disabilities: Individual level predictors and the role of the classroom peer context

**DOI:** 10.1371/journal.pone.0281598

**Published:** 2023-02-09

**Authors:** Eline Wagemaker, Verena Hofmann, Christoph M. Müller

**Affiliations:** 1 Department of Psychology, University of Amsterdam, Amsterdam, The Netherlands; 2 Research Priority Area Yield, University of Amsterdam, Amsterdam, The Netherlands; 3 Department of Special Education, University of Fribourg, Fribourg, Switzerland; University of Lisbon: Universidade de Lisboa, PORTUGAL

## Abstract

Prosocial behavior at school, such as helping and sharing, contributes to positive individual development, peer relations, and classroom climate. Students with intellectual disabilities (ID) may have difficulty to demonstrate prosocial behavior, but little is known about the levels of prosocial behavior and its predictors in this population. This study aims to describe the prosocial behavior of students with ID attending special needs schools and related individual (i.e., age, sex, and general functioning) and classroom level (i.e., classmates’ mean prosocial behavior) predictors. School staff members assessed prosocial behavior of 1022 students with ID (69.5% boys; *M*_age_ = 11.34 years, *SD* = 3.73, range: 4–19 years) at the beginning and the end of a schoolyear. We found that students with ID on average demonstrated moderate levels of prosocial behavior, this was lower compared to norms of typically developing students. Correlations within each timepoint proved that prosocial behavior was more present in older students, girls, and students with higher general functioning. Using a longitudinal multilevel model, we found that, prosocial behavior increased more over the schoolyear in older students and in students with higher general functioning. Classmates’ mean levels of prosocial behavior did not affect later individual prosocial behavior. We conclude that prosocial behavior in students with ID depends on several individual characteristics, but less on the levels of prosocial behaviors in their special needs classroom peer context.

## Introduction

At school, students can demonstrate many forms of prosocial behavior such as helping, sharing, comforting, supporting, cooperating, and showing respect for one another [1]. Such behaviors are important for individual development, promote positive peer relations among students, and benefit to a classroom climate that stimulates learning [2, 3]. Prosocial behavior is defined as voluntary actions intended to benefit others [3] and is influenced by both individual and contextual characteristics such as the classroom peer context [4, 5]. Students with intellectual disabilities (ID) may have difficulty to demonstrate prosocial behavior. These students are characterized by limitations in adaptive behaviors (i.e., conceptual, social, and practical everyday life skills; competence levels two standard deviations below population mean) and in intellectual functioning (i.e., IQ<70), with evidence of the disability before the age of 22 years [6]. The prevalence of ID is estimated to be between 0.05 and 1.55% [7]. Problems with social competencies, such as perspective taking [8, 9], may limit the prosocial behavior demonstrated by students with ID. Yet, little is known about prosocial behavior among these students. In many European countries, a large part of students with ID attend special needs schools [e.g., 86.6% in Germany and 80–99% in the Netherlands; 10, 11], making a study focusing on this context highly relevant to contribute to a better understanding of social development of students with ID. Therefore, the current study focuses on prosocial behavior of students with ID in special needs schools and its relation to a) individual level predictors (i.e., age, sex, and general functioning) and b) classmates’ prosocial behavior.

### Prosocial behavior in students with ID

Limited available literature suggests that there are only few studies on prosocial behavior considering individuals with ID. For example, one parent-report study showed that 260 children (6–12 years) with mild or moderate ID had close to average levels of prosocial behavior, but less than typically developing children [12]. Another study demonstrated that 98 adolescents with ID (11–15 years) themselves do not report significantly different levels of prosocial behavior compared to typically developing adolescents [13]. Nevertheless, their teachers only reported moderate levels of prosocial behavior, which was lower compared to the levels reported by adolescents with ID and their parents [13]. Relatedly, a study that only used teacher reports also found moderate mean levels of prosocial behavior in 44 children and adolescents (4–18 years) with mild to profound ID [14]. To our knowledge there is no teacher report study comparing students with and without ID on their prosocial behavior. Until now, results on prosocial behavior in ID are still mixed, with reports from parents and teachers suggesting that students with ID may have some difficulties to demonstrate prosocial behavior.

### Individual level predictors of prosocial behavior

#### Age

Children and adolescents have been proposed to show age-related increases in their prosocial behavior [see e.g., 3]. This development is supported by socio-cognitive developments such as perspective taking [15] as well as by motivational changes such as commitment to moral norms [15–17]. Only a few studies have assessed age effects in prosocial behavior in children and adolescents with ID. A cross-sectional study in students with ID (4–18 years) found no relation between age and prosocial behavior based on teacher reports [14]. One longitudinal parent-report study found that prosocial behavior of 555 children with ID generally increased over time (3–11 years), with steeper increases between 7 and 11 years [18]. During adolescence, prosocial behavior may even increase more [19]. As longitudinal studies in adolescents with ID are still lacking, the current study includes both children and adolescents with ID.

#### Sex

Typically developing girls have been perceived as more prosocial than boys [20]. Explanations for this sex difference center around socio-cognitive developments and socialization: girls develop moral reasoning, empathic concern, and perspective taking at an earlier age compared to boys and are also more socialized to show nurturance and caring behavior [21, 22]. For children and adolescents with ID, the findings on sex differences in prosocial behavior are similar: girls with ID demonstrate more prosocial behavior than boys with ID [12, 13]. However, there is one study which found no sex difference in teacher-reported prosocial behavior in 44 students with mild to profound ID [14].

#### General functioning

By general functioning, we mean the students’ level in their daily lives in areas such as conceptual, social, and practical functioning. Greater conceptual functioning (i.e., IQ) has been associated with more prosocial behavior [23]. Similarly, greater social functioning (i.e., perspective taking) has been linked to more prosocial behavior in children [see 15 for a meta-analysis]. In students with ID, it is still unclear whether general functioning and prosocial behavior are related. With regard to conceptual functioning, students with mild to moderate ID were not different from students with severe and profound ID in their prosocial behavior [14]. Also with regard to social functioning, the ability to empathize was not related to helping behavior in children with ID [24]. However, these two studies had small sample sizes (*N* = 44 and *N* = 28 respectively) and only focused on specific aspects of general functioning.

### Classroom peer context and prosocial behavior

Apart from individual level predictors, having classmates that demonstrate high levels of prosocial behavior may increase students’ individual prosocial behavior over time [5]. Such classroom effects are often explained by explicit peer influence mechanisms (e.g., when classmates provide compliments for prosocial behavior) or implicit peer influence mechanisms [e.g., the extent to which classmates display prosocial behaviors, which can create a classroom norm; for reviews see 25, 26].

To provide hints on how classmates affect prosocial behavior in students with ID attending special needs schools, we first reviewed the limited number of studies on susceptibility to peer influence in children and adolescents with ID [see also 27 for an overview]. These studies suggest a high susceptibility to peer influence in children and adolescents with ID in the domains of self-injurious behavior [28], challenging behavior [29], risky decision-making [30], social judgments [31], and positive mood [32]. We are aware of only one experimental study focusing on the effect of peer influence on prosocial behavior in adolescents with mild ID or borderline intellectual functioning [33]. In this study, adolescents first performed a donation game alone, then increased their prosocial behavior with virtual peer presence and increased again when the virtual peers provided feedback. Thus, there is some evidence for the short-term effects of peer influence on different kinds of behaviors in children and adolescents with ID outside the school context.

As a second step, we reviewed studies on longer term peer influence effects within the school context. We are only aware of two studies performed in special needs classrooms using the same dataset as the current study. For problem behavior, higher classroom levels of anxiety, problems relating socially, and communication disturbances predicted an increase of such problems in individual students with ID [34]. For adaptive behavior, students’ individual conceptual skills increased more when their classmates in special needs classrooms had greater conceptual skills [35]. It is still unknown whether such long-term classroom peer effects also exist for prosocial behavior.

### The current study

In the current study, school staff members in special needs schools reported on the prosocial behavior of their students at the beginning and the end of a schoolyear. We have three study goals. First, we aim to better describe the prosocial behavior of students with ID attending special needs schools. Besides investigating the mean level of prosocial behavior of students with ID, we compare their prosocial behavior to test reference norms. We hypothesize lower levels of prosocial behavior in students with ID compared to same-aged typically developing students [12–14]. On top of this, we descriptively assess the types of prosocial behaviors observed.

Second, we aim to investigate the role of individual level predictors in the frequency and development of prosocial behavior of students with ID. Current studies on individual level predictors of prosocial behavior are limited considering their cross-sectional designs and small sample sizes. Also, some mixed results exist. Therefore, we relate students’ age, sex, and general functioning to their prosocial behavior at both time points. We hypothesize higher levels of prosocial behavior in older students, girls, and students with greater general functioning [12, 13, 15, 18, 23]. Moreover, we explore whether these individual level predictors are related to the development of prosocial behavior over the schoolyear.

Third, we aim to establish whether classroom peer context effects exist for prosocial behavior in students with ID. We hypothesize that when students with ID have classmates that demonstrate high levels of prosocial behavior at the beginning of the schoolyear, this may increase their individual prosocial behavior at the end of the schoolyear [5, 33–35]. As prosocial behavior may be highly variant within special needs classrooms, we control for classroom heterogeneity in prosocial behavior [see 34 for a similar approach].

## Methods

### Participants

We adopted a longitudinal research design for this study with two timepoints (T1 and T2). The data were collected in Swiss special needs schools, providing classrooms for students with ID from childhood until adolescence. These schools can only be attended when a student receives a clinical diagnosis of ID, which is usually based on ICD-10 criteria and includes an assessment of intelligence (IQ < 70) as well as a clinical estimation of adaptive behavior levels. Students spend their entire school day (typically from about 8 am to 4 pm) in special needs schools and are assigned to lower grade (Kindergarten to Grade 2), middle grade (Grades 3–6), and upper grade (Grades 7–10) classrooms depending on their chronological age. While there are no additional formal rules for classroom assignment, combining students with many behavioral problems or very low adaptive competences in the same classroom is typically avoided.

Special needs schools were selected based on convenience sampling. We contacted 20 headmasters in the German-speaking part of Switzerland, 16 agreed to participate. In total, 1177 students attended these schools in 182 classrooms. We could not include all students due to decisions by parents or staff not to participate and due to missing data. Based on the available data, our sample included 1022 students who attended 176 classrooms. Thus, our sample was comprised of 86.8% of all students in the participating schools. The average number of included students per school was 63.88 (*SD* = 23.52, range = 25–116); classrooms contained on average 5.81 included students (*SD* = 1.53, range = 2–14). The mean age of included students at T1 was 11.34 years (*SD* = 3.73, range 4.17–19.08); 69.5% were male (710 boys and 312 girls). Information about students was provided by 352 school staff members (T1: 85.8% women; *M*_age_ = 46.71 years, *SD* = 11.42, range = 19–63). Most respondents had a special needs teaching diploma (57.0%); the remainders were regular teachers who provided instruction in specific school subjects, teaching assistants, therapists, or long-time teaching trainees. Participating staff members reported on average on 2.90 students (*SD* = 1.67, range = 1–8) whom they supervised daily at school. T1 measures were assessed between September and October 2018, T2 measures between April and June 2019. Assessments were conducted in German.

### Measures

#### Demographics

Staff members reported on students’ sex and age in months.

#### Individual prosocial behavior

At both time points, school staff members filled out the Prosocial Behavior subscale of the Strengths and Difficulties Questionnaire Teacher Report (SDQ; Goodman, Ford, Simmons, Gatward, & Meltzer, 2000 [36]). This subscale consists of 5 items regarding the extent of prosocial behavior (see Table 2 for all items). School staff members rated the items along a 3-point scale (0 = not true, 1 = somewhat true, 2 = certainly true). The outcome measure was the sum score ranging from 0–10. When scores on 3 or more items were missing, they were excluded from the analyses. When answers on 1 or 2 items missing, these scores were replaced by the mean score [based on scoring instructions, 37] before calculating the sum scores. The SDQ is valid and reliable in children and adolescents with ID [13]. In the current sample, internal consistency was also good at both time points (both α’s = .85).

#### Classroom prosocial behavior

The average classroom prosocial behavior was calculated as the mean of all students’ SDQ Prosocial sum scores at T1 per classroom. Classroom heterogeneity was calculated as the standard deviation of the average classroom prosocial behavior, with a greater standard deviation in a classroom signifying more heterogeneity of prosocial behavior levels in this classroom.

#### General functioning

We operationalized students’ general functioning by assessing their adaptive behavior level using a German teacher version of the Adaptive Behavior Assessment System-3 [ABAS-3; 38]. It describes 174 competencies in the domains of conceptual, social, and practical skills, the mastery of which is assessed on a scale from 0 (not able to do this behavior) to 3 (always or almost always when needed). A General Adaptive Composite (GAC) score was determined, higher scores indicated more competencies. The ABAS-3 demonstrates adequate validity and reliability [interrater reliability r = .72-.81; α = .86-.99; 38] and very high internal consistency in the current sample (α = .99 at T1 and T2).

### Procedure

The current study was approved by the Research commission of the Department of Special Education at the University of Fribourg (with reference to the Ethical Principles of Psychologists and Code of Conduct by APA and the Declaration of Helsinki by WMA). Recruitment of participating schools was based on written information about the study and personal meetings with school administrators. Information on students was collected completely anonymously. Researchers never had access to the names of school staff, parents, or students. Therefore, it was impossible to identify individuals.

Parents received a letter from school in which they were informed about the study, its anonymous nature, and the fact that no data on medical diagnoses were collected. The letter was submitted also in simple language and in nine frequently spoken languages in Switzerland. As accepted by the Research commission evaluating the study design and ethical requirements, participation was voluntary and parents could inform the responsible class teacher when they did not wish that teachers provided information on their child (i.e., written passive consent). School employees were also informed about the study and could refuse to participate.

An anonymous coding system was used for data management. In each school, research assistants distributed paper-pencil questionnaires for every participating student to staff members as part of a meeting at the first and the second measurements. Questionnaires were filled out during this meeting and/or privately and took about 30 minutes. Staff members received no personal rewards besides detailed feedback on the study results later.

### Statistical analyses

#### Descriptive analyses

To describe prosocial behavior, we calculated the whole samples’ mean sum scores at the beginning (T1) and the end (T2) of the schoolyear. The distribution of scores was interpreted based on cut-points for typically developing 4- to 17-year-olds [37]. We tested whether our scores were lower than those of the test reference norms of British typically developing 5- to 15-year-olds [39] using an online independent *t*-test calculator [40]. Effect sizes were calculated using Cohen’s *d*, which can be interpreted as small (.2), medium (.5) or large [.8; 41]. Finally, we also checked the types of prosocial behavior based on item scores at T1 and T2. We also computed Pearson correlations between all study variables. Effect sizes for these correlations were interpreted as small (*r* = .1), medium (*r* = .3) or large [r = .5; 41]. We expected that individual prosocial behavior at T1 and T2 was positively correlated to age and general functioning, and negatively correlated to sex (reference female).

#### Longitudinal multilevel models

To investigate changes from T1 to T2, multilevel longitudinal models in Mplus Version 8 were fitted [42]. These analyses were performed in a two-level framework, and we allowed the intercepts to vary at Level 1 (students) and Level 2 (classrooms). To explore the overall time difference regardless of sex, age, or adaptive behavior, we first fitted a simple model with a Wald test (similar to a dependent *t*-test, but here in a multilevel framework). Second, we explored longitudinal effects in the individual level predictors by predicting individual prosocial behavior at T2 from students’ sex, age, and adaptive behavior, controlling for individual prosocial behavior at T1 (Model 1). Third, the of the classroom peer context was tested. We included the classroom mean of all students’ T1 scores as a predictor, while again controlling for individual prosocial behavior at T1. This is a common method for examining classroom context effects [43]. In addition, we controlled for classroom heterogeneity of prosocial behavior at T1. We fitted a similar model as before with classroom mean and heterogeneity of prosocial behavior as extra predictors (Model 2). We expected that a higher classroom mean prosocial behavior at T1 predicted higher scores on individual prosocial behavior at T2, controlling for individual prosocial behavior at T1, age, sex, adaptive behavior, and classroom heterogeneity at T1.

## Results

### Preliminary analyses

For 25 students, responses on one or two items were missing on the SDQ Prosocial Behavior subscale at T1 or T2. These were therefore replaced by the mean score before calculating the sum scores. Available data on adaptive behavior (*N* = 922) showed that the mean ABAS-3 GAC score was 45.97 (*SD* = 23.35) and that 46.9% of the sample could be described as extremely low, 20.8% as low, 23.2% as below average, and 9.1% as at least average in general functioning.

### Descriptive analyses

At T1, mean sum prosocial behavior was 5.50 (*SD* = 2.83) and at T2, mean sum prosocial behavior was 5.76 (*SD* = 2.79). This shows that, compared to the scale range from 0 to 10, prosocial behavior levels were moderate. Compared to the SDQ cut points, 53.5% (T1) and 55.6% (T2) of the students had close to average prosocial behavior, 12.5% (T1) and 13.6% (T2) had slightly lowered scores, 9.5% (T1) and 8.8% (T2) had low scores and 24.5% (T1) and 22% (T2) had very low scores, suggesting great variability between students. As expected, students with ID on average had significantly lower prosocial behavior at both timepoints than norms of typically developing students (*M* = 7.2, *SD* = 2.4; both *p’s* < .001), demonstrating medium effects. The scores per item are shown in Table 1. Students scored relatively lowest on item 5 (i.e., providing voluntary help) and relatively highest on item 3 (i.e., being kind to younger children).

**Table 1:** Item means (M), standard deviations (SD) and percentages on the SDQ prosocial subscale at both timepoints (T1 and T2).

	T1					T2				
	*M*	*SD*	Not true	Somewhat true	Certainly true	*M*	*SD*	Not true	Somewhat true	Certainly true
**1. Likes to share with other children (good, games, pens, etc.)**	.95	.70	27.4%	50.6%	22%	1.00	.68	23%	53.7%	23.3%
**2. Is considerate towards others**	1.13	.69	18%	50.9%	31.1%	1.17	.68	15.9%	51.1%	33%
**3. Is kind to younger children**	1.41	.69	11.5%	35.8%	52.7%	1.44	.67	10.2%	35.6%	54.2%
**4. Is helpful if someone is hurt, upset, or feeling ill**	1.18	.76	21.5%	38.7%	39.8%	1.23	.75	19.4%	38.3%	42.4%
**5. Often helps others voluntarily (parents, teachers, children)**	.83	.71	35.3%	45.9%	18.8%	.91	.74	32.2%	44.6%	23.2%

Abbreviations: SDQ: Strengths and Difficulties Questionnaire.

Item range: 0 = Not true, 1 = Somewhat true, 2 = Certainly true.

Table 2 shows the correlations between the study variables. All variables were significantly positively or negatively correlated, except for sex with T1 classroom heterogeneity and age. As expected, individual and classroom prosocial behavior scores were higher for older students (medium effect sizes), girls compared to boys (small effect sizes), and students with higher levels of adaptive behavior (medium to large effect sizes).

**Table 2:** Correlations between the key variables.

	**2**	**3**	**4**	**5**	**6**	**7**
**1. T1 individual prosocial behavior**	.728**	.538**	-.096**	.246**	-.124**	.591**
**2. T2 individual prosocial behavior**	–	.402**	-.101**	.245**	-.107**	.507**
**3. T1 classroom prosocial behavior**		–	-.179**	.416**	-.087**	.352**
**4. T1 classroom heterogeneity**			–	-.12**	.00	-.099**
**5. T1 age**				–	-.037	.068*
**6. Sex (reference female)**					–	-.067*
**7. T1 adaptive behavior**						–

*p < .05;

** *p* < .001.

### Longitudinal multilevel models

The Wald test used to test the overall time difference indicated no significant change in individual prosocial behavior from T1 to T2 (*p* = .833), indicating that prosocial behavior was relatively stable over the schoolyear. The results for the longitudinal models incorporating the predictors are depicted in Table 3. The results of Model 1 showed that T1 individual prosocial behavior significantly predicted T2 individual prosocial behavior, demonstrating a large effect (β = .630). This again indicates that prosocial behavior was moderately stable over time. We also found small main effects of age (β = .084) and adaptive behavior (β = .140) on T2 individual prosocial behavior, controlling for T1 prosocial behavior. This indicates that individual prosocial behavior increased more over time in older students and in students with higher levels of adaptive behavior. There was no significant main effect of sex on later individual prosocial behavior. We ran Model 2 to test our classroom peer context hypothesis. Mean classroom prosocial behavior at T1 did not significantly predict the change in individual prosocial behavior (β = -.201). The control variable classroom heterogeneity was also not significant (β = -.143). The pattern of results for the individual level predictors was highly similar to Model 1.

**Table 3:** Unstandardized results from longitudinal models predicting T2 prosocial behavior based on T1 individual variables and classroom prosocial behavior.

	Model 1	Model 2
	*B* (*SE*)	*B* (*SE*)
**Male**	-.210 (.148)	-.214 (.147)
**Age**	.062 (.021)*	.069 (.023)*
**Adaptive behavior**	.016 (.004)**	.017 (.004)**
**Individual T1 prosocial behavior**	.614 (.030)**	.625 (.031)**
**Variance level 1**	3.229 (.208)**	3.301 (.208)**
**Classroom prosocial behavior T1**		-.064 (.064)
**Classroom heterogeneity T1**		-.080 (.092)
**Variance level 2**	.250 (109)*	.238 (.109)*

*p < .05;

** *p* < .001.

## Discussion

The goal of this study was to describe the prosocial behavior of students with ID attending special needs schools and its relation to a) individual level predictors (i.e., age, sex, and general functioning) and b) classmates’ mean prosocial behavior. We investigated this in a large sample of students with ID based on staff member reports at the beginning and the end of the schoolyear.

### Prosocial behavior of students with ID

Students with ID demonstrated moderate levels of prosocial behavior, which was in line with earlier studies using teacher reports of prosocial behavior in students with ID [13, 14]. As expected and suiting parent report results [12], this was lower than what is found in typically developing students. Additionally, we gained information on the frequency of different types of prosocial behavior observed. In terms of descriptives, we found that being helpful when somebody is hurt, being considerate to others’ feelings, and being kind to younger children were more often observed than sharing with other children and helping others voluntarily. This fits with earlier research on the requirement of more complex social-cognitive skills (e.g., perspective taking) for display of certain types of behavior, which may be limited in children and adolescents with ID [8, 9, 15–17] and can potentially explain the observed patterns of prosocial behavior in students with ID.

### Individual level predictors of prosocial behavior

#### Age

Our cross-sectional analysis using a broad age range (4–19 years) showed that higher students’ age was associated with more prosocial behavior. This was in line with our expectation based on a longitudinal study on prosocial behavior in children with ID [18]. However, it was not in line with a cross-sectional study [14] on students with ID (4–18 years) showing no correlation between age and prosocial behavior, which could be due to limited power in the analyses of this study related to a small sample size. The increase in prosocial behavior with age could be explained by increases in socio-cognitive developments with age [16], which have also been found in children and adolescents with ID [44].

Our longitudinal analysis showed that prosocial behavior remained relatively stable over the schoolyear. This suggests that for students with ID these types of behaviors are difficult to develop and fits with the other longitudinal study on prosocial behavior in children with ID only demonstrating an increase when prosocial behavior was assessed over a longer time period [3–11 years, 18]. Interestingly, we found that prosocial behavior developed more in older students. This can be linked to literature describing adolescence as a developmental period with an increasing need to contribute to society as well as to develop prosocial goals [19]. To better understand the development of prosocial behavior in students with ID, future longitudinal studies over longer time periods than considered here are critical, especially in the adolescence period.

#### Sex

We found that girls with ID demonstrated more prosocial behavior at school than boys with ID, which was in line with our expectation and earlier research [12, 13, 20]. Our result was not in line with, to our knowledge, the only different study in the school context showing no sex difference in prosocial behavior between boys and girls with ID [14]. This could be explained by the sex difference being small and therefore only being detected in a large sample. Explanations about girls demonstrating earlier socio-cognitive development and being socialized to show more caring behavior than boys [21, 22] may thus also apply to students with ID. Our longitudinal analysis demonstrated no sex difference in the development of prosocial behavior over the schoolyear. Potentially this could be due to the relatively short timespan of our study. To further disentangle potential sex differences in the development of prosocial behavior of students with ID, we recommend more extensive longitudinal research.

#### General functioning

Until now, studies using smaller samples were not able to find a relation between general functioning aspects and prosocial behavior in students with ID [14, 24]. However, our study using a broad indicator of general functioning and a large sample demonstrated that students with ID with greater general functioning display more prosocial behavior, which is in line with studies on typically developing individuals [15, 23]. Also, students with greater general functioning increased more in their prosocial behavior over the schoolyear. Indeed, prosocial behavior requires socio-cognitive competencies that are also part of general functioning [16]. General functioning may therefore provide students with more opportunities to show and develop prosocial behavior. More research is necessary to disentangle relations between specific aspects of general functioning and prosocial behavior.

### Classroom peer context and prosocial behavior

Classroom average prosocial behavior did not affect later individual prosocial behavior of students with ID. This is not in line with our expectation based on an earlier study showing such classroom effects in typically developing students [5]. Nevertheless, the classroom peer context effect was only small in this study and another study including less classrooms also did not find a such an effect on prosocial behavior [45], suggesting that larger samples may be necessary to detect this effect. Until now, only few peer influence studies have been performed in children and adolescents with ID, suggesting some long-term classroom peer effects on problem and adaptive behavior [34, 35] as well as short-term peer influences on prosocial behavior [33]. Based on our results, it remains an open question whether students with ID are not able to perceive classroom norms on prosocial behavior or have less competencies or less motivation to demonstrate prosocial behavior when stimulated by classmates.

### Strengths and limitations

The current study has several strengths. First, by using anonymous staff member reports we were able to assess information on the greatest part of students attending the participating special needs schools. This allowed us to study both individual and classroom predictors on prosocial behavior. Second, our longitudinal design created the possibility to study predictors of the development of prosocial behavior over the schoolyear. Including both individual and classroom peer context variables is a relatively new approach in research on ID, revealing new insights on the social development of students with ID. Third, as many studies on the ID population focus on problem behavior or risk factors, our focus on prosocial behavior provides important leads for positive development in students with ID.

Despite these strengths, we acknowledge a few limitations of this study. First, we solely relied on staff member reports. Although this allowed us to recruit a large sample also including students with low levels of general functioning, it limits the studied prosocial behavior to what is seen and memorized by staff. Classroom or schoolyard observations could provide a deeper view on prosocial behavior in students with ID, which may especially be helpful to unravel underlying mechanisms or peer influences. Second, we assessed only five types of prosocial behaviors. Prosocial behavior is a broad construct ranging from sharing to cooperating and several subtypes could have different developmental trajectories [3, 16]. We therefore encourage future research to study the development of a broader range of prosocial behaviors in students with ID. Third, we operationalized general functioning by adaptive functioning and did not gather information on other indicators such as IQ or comorbid disorders. Although students entering special needs schools must have a clinical diagnoses of ID, additional information on IQ or comorbid disorders based on school reports could be added in future studies. Fourth, earlier research as well as this study on prosocial behavior in individuals with ID is mostly conducted in Western countries. As cultural norms may provide interesting nuances in the prosocial behavior displayed [46], we acknowledge that our hypotheses and conclusions may not always be applicable to countries with other cultures.

## Implications and conclusions

We conclude that students with ID demonstrate prosocial behaviors such as sharing, being considerate, and helping. However, such behaviors are less frequently observed than in typically developing students. Furthermore, prosocial behavior in students with ID appears to be more dependent on individual characteristics, such as age, sex, and general functioning, than on the level of prosocial behaviors exhibited in their special needs classroom peer context. A next step in research could be to investigate interactions between these predictors. By this, subgroups of students with ID that demonstrate low prosocial behavior could be targeted more specifically. For now, our results emphasize the importance of individual level predictors in the prosocial behavior of students with ID, which implies the relevance of socio-cognitive competencies for demonstrating prosocial behavior. We therefore recommend future research to select instruments to assess competences such as perspective taking, empathy, and acting along personal norms. By also adding more timepoints, it could be established whether such competences mediate the relation between individual predictors and the development of prosocial behavior. Finally, it should be noted that we focused on just one classroom variable (i.e., classmates`levels of prosocial behavior). As there are further classroom characteristics, such as density of peer relationships and teacher support [see e.g., 47], future research may also consider these factors as predictors of prosocial behavior.

To further support prosocial behavior in students with ID, we encourage the development of intervention programs specialized for students with ID. First studies in related areas have already been conducted, such as a theory of mind intervention which can effectively increase socialization of students with ID [48]. Research in typically developing students has shown that promoting prosocial behavior via school-based interventions or mentors at school could be effective [49, 50]. For students with ID attending special needs schools, intervening on the general prosocial behavior levels of classmates may be less effective, but more research on short-term effects of the classroom context on prosocial behavior is necessary. Supporting prosocial behaviors in students with ID attending special needs schools could have great benefits for their individual development, peer relations, as well as for classroom climate [2].
